# Managing medicines at the end of life: a position paper for health policy and practice

**DOI:** 10.1108/JHOM-11-2020-0440

**Published:** 2021-11-18

**Authors:** Asam Latif, Christina Faull, Justin Waring, Eleanor Wilson, Claire Anderson, Anthony Avery, Kristian Pollock

**Affiliations:** School of Health Sciences, University of Nottingham , Nottingham, UK; LOROS Hospice Leicester , Leicester, UK; Health Services Management Centre, University of Birmingham , Birmingham, UK; School of Pharmacy, University of Nottingham , Nottingham, UK; School of Medicine, University of Nottingham , Nottingham, UK

**Keywords:** Palliative care, Community pharmacy, End-of-life

## Abstract

**Purpose:**

The impact of population ageing is significant, multifaceted and characterised by frailty and multi-morbidity. The COVID-19 pandemic has accelerated care pathways and policies promoting self-management and home-based care. One under-researched area is how patients and family caregivers manage the complexity of end-of-life therapeutic medicine regimens. In this position paper the authors bring attention to the significant strain that patients and family caregivers experience when navigating and negotiating this aspect of palliative and end-of-life care.

**Design/methodology/approach:**

Focussing on self-care and organisation of medicines in the United Kingdom (UK) context, the paper examines, builds on and extends the debate by considering the underlying policy assumptions and unintended consequences for individual patients and family care givers as they assume greater palliative and end-of-life roles and responsibilities.

**Findings:**

Policy makers and healthcare professionals often lack awareness of the significant burden and emotional work associated with managing and administering often potent high-risk medicines (i.e. opioids) in the domiciliary setting. The recent “revolution” in professional roles associated with the COVID-19 pandemic, including remote consultations and expanding community-based care, means there are opportunities for commissioners to consider offering greater support. The prospect of enhancing the community pharmacist's medicine optimisation role to further support the wider multi-disciplinary team is considered.

**Originality/value:**

The paper takes a person-focused perspective and adopts a holistic view of medicine management. The authors argue for urgent review, reform and investment to enable and support terminally ill patients and family caregivers to more effectively manage medicines in the domiciliary setting. There are clear implications for pharmacists and these are discussed in the context of public awareness, inter-professional collaboration, organisational drivers, funding and regulation and remote care delivery.

## Introduction

The commissioning and delivery of palliative care is inconsistent even in high-income countries and it is estimated that only 14% of people judged to be in need currently receive it ( Etkind *et al.* , 2017 ). For patients and family caregivers, the pressures of managing serious illness including dementia and/or frailty are escalating and projected to rise significantly in the coming decades ( Sleeman *et al.* , 2019 ). Effective deployment, use and administration of medicines to deliver optimal relief of distressing symptoms is at the heart of effective palliative care and critical for enabling dying patients to remain at home. For these patients, end of life care depends on effective use of prescribed medication to manage common and sometimes unpredictable symptoms including pain, nausea, delirium and breathlessness ( NICE, 2020 ).

The COVID-19 pandemic has had a substantial impact and advanced care delivery pathways. The effort to enable social distancing and reduce pressure on the NHS has led to increases in remote and e-Health technologies. The longer-term impact of these changes on the delivery of care to older people and to vulnerable groups is unknown. However, they have inadvertently quickened the gradual shift towards policies promoting patient self-management of chronic and terminal diseases ( Lederle and Bitzer, 2019 ). Consequently, expectations to self-manage care have become more prevalent. Family caregivers are also being encouraged to adopt work analogous to the complex tasks traditionally performed by health professionals including assessments, monitoring and judging when and how to act to manage symptoms and side effects ( Antunes *et al.* , 2020 ).

As well as drug administration, the associated work of medicines management is often underestimated and has recently become more complicated. New care pathways and remote prescribing practices contribute to this complexity. As co-morbidities and the number of medicines used by older people continue to increase, there will be a greater emphasis on resolving issues including non-adherence, adverse effects and problematic polypharmacy. The question arises as to how the healthcare system can support self-management and, more specifically, who may be best placed to offer this help. In this position paper we identify some of the unintended consequences of palliative and end-of-life medicine management in the domiciliary setting. Our principal aim is to give voice to patients and family caregivers, expose the burdens placed on them when managing complex medicines regimes. Finally, we offer insight into the potential for community pharmacy to assume a greater professional role.

## The shifting care landscape: consequences for patients and families

Before the onset of the pandemic, policy approaches encouraging the public to become more “informed” or “experts” in self-managing their illness were principally seen to be in line with agendas promoting autonomy and choice ( Vadiee, 2012 ). Commitments to enable patients to die at home are largely seen to be in step with expectations that this is the preferred place and choice of the majority ( Gomes *et al.* , 2011 ). It is assumed that patients and their family caregivers would be willing to engage in self-management to enable better forward planning and the eventual “good death”. Shifting the place of death from hospital to home is also an arrangement that is economically less costly ( McCaffrey *et al.* , 2015 ). In the UK, the NHS long term plan advocates out-of-hospital care by promoting a contemporary model of self-care and management to support proactive, personalised and well-coordinated care for all people in their final year of life ( NHS England, 2019 ).

However, concerns have been raised that the home is increasingly becoming encroached upon, becoming “annexed” as an extension of the hospital. The promotion of norms about managing end-of-life care at home without adequate examination of the unintended adverse consequences could result in constraining rather than enabling patient “choice” ( Pollock, 2015 ; Milligan *et al.* , 2016 ). While some level of self-management may be desirable during the course of an illness, end-of-life care poses different and complex challenges that often require professional support. Patients will often become too ill to manage care themselves and family caregivers may also have their own frailties and comorbidities. Mair and May (2014) aptly reframe the context from asking how over-stretched healthcare systems can cope with rising demands from patients to asking how overburdened patients and family caregivers are able to cope with the unrelenting demands imposed on them by an overly complex, fragmented and bureaucratic system.

The pandemic has further impacted on how palliative and end-of-life care is managed. Social isolation, fear of contracting COVID-19 and caution not to overburden the NHS have all determined the way care is perceived, accessed and delivered. There is also increased pressure on family caregivers to assume greater responsibility for decisions about administering medicines in cases where healthcare professionals may be absent ( NICE, 2020 ). If policies promoting self-management at home are not carefully governed, the delivery of care can quickly become burdensome for both patients and especially family caregivers. The escalation of eHealth services has also quickened, offering efficient, convenient and safe means for patients to access health and medicines advice. For example, the adoption of telemedicine to remotely monitor health and support self-care is now largely seen as a means to avoid cross-contamination between patients and front-line health workers; consequently telemedicine is becoming a widespread alternative to face-to-face consultations ( Bokolo, 2021 ). The drawback of these new advancements may risk excluding people from minority groups, those who are not “user ready” or who may be digitally disadvantaged in other ways ( Sutherland *et al.* , 2020 ).

There remains an underestimation of the support needed for patients and family caregivers to effectively self-manage care and COVID-19 has added to this burden. The managing medicines study ( Pollock *et al.* , 2021 ) reinforced earlier findings that it is often the day-to-day practical problems associated with medicine use that lead to unintentional non-adherence. Concerns and anxiety can also arise around poor symptom control and administering potent medicines (e.g. opioid controlled drugs) ( Wilson *et al.* , 2018 ). The study also raises questions about what can reasonably be expected of patients and family caregivers in managing palliative and end-of-life care medicines and highlights the need for greater professional understanding and support ( Box 1 ). System-wide review, reform and investment is needed to protect those who are most vulnerable. They are least likely to resist self-management discourses or possess the social or economic capital required for self-management of complex treatment regimens at home. However, reducing this burden of care will require far reaching changes to service organisation and delivery and may also impact on (inter) professional roles and boundaries. One option could be to consider contracting extended services from community pharmacists who largely remain an under-used resource and who could assume greater roles in palliative medicine management services ( O'Connor *et al.* , 2011 ; Latif *et al.* , 2020 ).

Box 1 **Overview of findings from the “Managing medicines for patients with serious illness being cared for at home” study.**

This qualitative investigation explored how patients, their family caregivers and health care professionals engage in the tasks of managing complex medication regimens for patients with severe and terminal illness.

1. Patients and families often engaged in demanding and complex “work” in managing medications at the end of life. This work involved practical, physical and emotional components. It was organised in terms of space and place and embedded within the goals and activities involved in carrying on with normal life. Others have also highlighted the problem of delayed access to medicines via community pharmacies due to unavailability of stock and lack of coordinated communication between the pharmacy, health professionals and patients ( Ogi *et al.* , 2021 ).
2. There was often little professional engagement, guidance or information on how to manage the practical aspects of medicines management or understanding of the problems and concerns that these could pose.
3. As the patient's health deteriorated family caregivers increasingly assumed the role of care coordinator, managing all aspects of medicines management. Participants described the need to maintain vigilance about the medicines prescribed for their relative, especially when changes were made by different professionals who lacked knowledge of the case. Managing complex medicines in the home added to an already substantial workload of organising other aspects of palliative care and so further depleted patient and family caregivers' physical and emotional resources.
4. Palliative, health and social care arrangements were experienced as complex and often fragmented or poorly coordinated. This was particularly problematic during the later stages of the patient's illness, when deterioration could be rapid with frequent changes to symptom burden and prescribed medication.
5. Establishing a relationship with a key professional who could help families negotiate the health care system had a significant positive impact on experience of care.
6. Patients and family caregivers were often unaware of the help and support the pharmacist was able to provide. It was clear the potential for pharmacists to help patients optimise medicines was not being fully realised.

## A case for greater community pharmacist involvement

In England, public access to the 11,500 community pharmacies is good with approximately 90% being located within a twenty-minute walk of where people live ( Todd *et al.* , 2014 ). Pharmacists provide a range of professional services including medication reviews ( Latif *et al.* , 2017 ), advise on healthy living ( Nazar *et al.* , 2019 ) and services that promote self-care ( Anderson and Sharma, 2020 ). During the pandemic, pharmacies saw a rise in the numbers of people accessing their services as the public struggled or were reluctant to access other healthcare providers such as GPs and hospitals. In this time, pharmacists' have shown innovation, adaptability and flexibility to assume new roles. This has included accommodating COVID-19 vaccinations, which has been particularly evident in response to the unique challenges that the pandemic lockdown posed ( Cadogan and Hughes, 2021 ). In addition, the pandemic has exposed deeper inequalities with marginalised groups being disproportionally affected by COVID-19 ( Bambra *et al.* , 2020 ). Others have highlighted that pharmacy provision follows a “positive care law” where a greater number of pharmacies are found in areas of highest deprivation ( Todd *et al.* , 2014 ). The greater access in such areas means there is potential for pharmacist to play a valuable part in promoting and delivering a more equitable service.

In addition to their existing roles, pharmacists could further contribute to enhancing palliative care services by building system capacity and resilience (i.e. Safety-2 harm reduction approaches) ( Hollnagel *et al.* , 2015 ). This could be realised through coordinating and advising on medicine optimisation and administration (including anticipatory medicines) and brokering the gaps between professions around medicine prescribing, deprescribing and use ( Wilson *et al.* , 2020 ). For example, palliative domiciliary medication reviews could be made available for those unable to travel to the pharmacy or via “telemedicine” to those hesitant or unable to attend in person ( Calton *et al.* , 2020 ). There are already promising pharmacist-led palliative care support services emerging. Good examples of these include the Rural Palliative Care Pharmacist Practitioner project ( Akram *et al.* , 2017 ) and medicines optimisation services for patients with advanced cancer pain ( Edwards *et al.* , 2019 ) ( Table 1 ). Benefits may also be seen with initiatives that seek to upskill community pharmacy support staff as observed in the evaluation of the “MacMillan pharmacy service” ( Bennie *et al.* , 2015 ).

For patients and family caregivers, an assigned pharmacist as a single point of contact may hold significant benefits. This may include dedicated support for patients and families in managing complex medicines regimes and help in navigating a complex and sometimes bureaucratic system of care. A striking finding from the managing medicines study was the notable lack of patient, family caregiver and professional awareness and use of the community pharmacist's knowledge, skill sets and ability to provide advice and support for medicine-related issues or problems ( Pollock *et al.* , 2021 ). Even established NHS funded medication review services that aim to improve patients' knowledge of medicines and use ( Latif *et al.* , 2017 ) did not feature as strongly as one may have hoped. Therefore, alongside service development and reconfiguration, comprehensive public awareness strategies and campaigns are needed to showcase pharmacist roles and responsibilities to foster “help-seeking” behaviours. The initial aim should be for all patients and family caregivers to be able to be aware of and be able to easily access expert help and advice on medicines management and symptom control whenever they feel this is necessary.

## Overcoming barriers: realising the vision

### Public awareness

As mentioned, by and large, public awareness of the potential for community pharmacy services to provide medicine support, beyond supplying dispensed medicines and managing minor ailments, remains low ( Hindi *et al.* , 2018 ). Nevertheless, there is evidence that pharmacists are able to significantly help patients minimise harm from high-risk medicines. Examples include reducing the opioid load, improving symptom management, promoting risk-mitigation strategies and reducing adverse clinical outcomes ( Jordan *et al.* , 2021 ). Concerted efforts are needed to raise patient, caregiver and professional awareness and expectations to promote pharmacists' skills in managing more than simple minor ailments ( Mossialos *et al.* , 2015 ; Tait *et al.* , 2013 ). However, care is needed not to overpromise and under deliver. Future research should therefore explore the variability in pharmacy's preparedness and willingness to deliver such professional support, their capacity to implement services and how this may be constrained by factors such as existing workload, organisational and contractual barriers.

### Inter-professional collaboration

With a growing number of pharmacists in the UK becoming independent prescribers, pharmacists are well placed to improve safety, manage medicines and alleviating GP workforce pressures ( Stone and Williams, 2015 ). Reconfiguring care to enable an increased contribution of pharmacists will require collaboration including discussions around a more comprehensive shared access of medical records between different professional groups. Despite the good progress made in integrating GP-practice based pharmacists within the wider multi-disciplinary team, barriers for the community pharmacist remain and include factors such as the historical power imbalance in GP-pharmacist relations, differing contractual goals and pharmacists working remotely in relative silos ( Waring and Latif, 2018 ). Factors that have been shown to encourage interprofessional collaboration include early-career co-education to foster understanding of each other's skills and knowledge and utilising compatible technologies to facilitate communication between professional groups ( Bollen *et al.* , 2019 ). Again, further research is needed and should seek to explore how such measures could impact on GPs and pharmacist collaboration, how they may organise work and how patients can be a full partner in any such arrangement.

### Organisational drivers, funding and regulation

Joint contractual arrangements and “quality-driven incentives” have been proposed to promote pharmacist integration within the patient's primary care pathway ( Hindi *et al.* , 2019 ). “Primary Care Networks” (PCNs) offer an opportunity for policy makers and commissioners to acknowledge, integrate and extend community pharmacist's roles to fully optimise medicine support to patients and caregivers. These networks are assuming contractual responsibilities and promoting integrated care. The NHS, local authorities, voluntary and community organisations will be working together to take responsibility for the resources and health of a population in a defined area ( Anderson and Sharma, 2020 ). With appropriate funding, strategic vision and motivation, substantial change is possible. Furthermore, NHS leaders in England are pursuing changes to the healthcare system, structures and legislation via new “integrated care systems” (ICSs) ( NHS England and NHS Improvement, 2020 ). While the proposals do offer hope for a better coordinated service between health and care organisations, concerns have been expressed as to whether the timing of these proposals will help or hinder the recovery from the pandemic ( Alderwick *et al.* , 2021 ). Further investigation is needed to evaluate the implications of reorganisation, how the proposed changes will work in practice and the extent to which this will improve the patient/caregiver experience.

### Remote care: eHealth

Another opportunity that emerged during the pandemic was the ways information technology was better deployed to engage with and gather patient data outside traditional healthcare settings. Going forward, a full assessment is needed to fully take advantage of these remote eHealth strategies. Reconfiguration of existing care models may be needed including implementation and training for professionals and policy ambitions to use this technology proactively rather than reactively to achieve sustained benefits in the long-term ( Smith *et al.* , 2020 ). Social, technological and organisational factors should be considered to overcome barriers to accessing support and through this reduce the burden of self-care at a distance ( Bokolo, 2021 ). Pockets of progress can be seen in Wales where funding streams for specialist palliative care services have become more evident resulting in service innovations. Practically this includes 24-h access to a specialist consultant offering telephone advice, pharmacy services to improve access to medicines and funding to support professional education ( Welsh Government, 2013 ). Future research in this area should seek to assess public and professional acceptance of eHealth, implementation, effectiveness and cost-effectiveness of community pharmacist-led palliative medicine management models.

## Conclusion

Before the pandemic, commentators suggested the delivery of care to the elderly was already at a crisis point and heading towards the “brink of disaster” ( Dann, 2014 ). The COVID-19 pandemic has posed new challenges but has also led to innovations in the way healthcare is organised and managed. With an ageing population, there will continue to be growing demands for palliative and end-of-life care with mounting pressures on primary, secondary and acute care services. A “left shift” in expectations (moving care downstream into the community) will inevitably lead to patients and their families assuming greater responsibility for self-care. Effective medicines management will always be the cornerstone to enable care to be optimised for serious illness and at the end-of-life. It is currently unknown how the legacy of the pandemic will impact on the long-term organisation and delivery of care. We acknowledge that this is a limitation of our understanding. What should be central to any further research and debate is a greater understanding of the extent to which patients and family caregivers are willing or even able to self-manage complex medicine regimens and how professionals can best support them.

In the UK, and elsewhere, community pharmacy services are expanding and the paper suggests clear implications for pharmacists. Considering the additional challenges the pandemic has brought, we call for urgent review, reform and investment to support greater involvement of pharmacists in medicine management for patients approaching the end-of-life. This underlines the considerable scope and potential for contracting pharmacists as part of the multi-disciplinary team to support medicine optimisation and counselling that could help patients and family caregivers ( Nadeem *et al.* , 2021 ). With appropriate remuneration, organisational support and public awareness campaigns, the community pharmacist is well positioned to provide much needed support for pharmaceutical care to patients approaching the end of life.

## Figures and Tables

**Table 1:** Examples of service innovations extending pharmacists palliative care role

Community pharmacy service	Brief details of the intervention
MacMillan Rural Palliative Care Pharmacist Practitioner (MRPP) project ( Akram *et al.* , 2017 )	• Better professional integration of the MRPP in different care settings and teams and promote awareness of the clinical skill set of pharmacists • Development of medicine related educational resources for other health and social care professionals • Point of contact for advice on sourcing medicines • Bespoke training on pharmaceutical matters for care home staff
Community pharmacist medicines optimisation service for patients with advanced cancer pain ( Edwards *et al.* , 2019 )	• Delivery of one patient-pharmacist face-to-face or two telephone “medicines optimisation” consultation(s) (∼20–30 min) • Format of the consultation was based on the community pharmacy Medicine Use Review (MUR)/New Medicine Service (NMS) • Advice to manage medicine-related problems • Signposting and referral to other healthcare professionals when needed
